# Screening of the Supercritical Impregnation of *Olea europaea* Leaves Extract into Filaments of Thermoplastic Polyurethane (TPU) and Polylactic Acid (PLA) Intended for Biomedical Applications

**DOI:** 10.3390/antiox11061170

**Published:** 2022-06-14

**Authors:** Noelia D. Machado, Cristina Cejudo-Bastante, María L. Goñi, Nicolás A. Gañán, Lourdes Casas-Cardoso, Casimiro Mantell-Serrano

**Affiliations:** 1 Instituto de Investigación y Desarrollo en Ingeniería de Procesos y Química Aplicada (IPQA-UNC-CONICET), Av. Vélez Sarsfield 1611, X5016GCA Córdoba, Argentina; nmachado@unc.edu.ar (N.D.M.); laura.goni@mi.unc.edu.ar (M.L.G.); nicolas.ganan@unc.edu.ar (N.A.G.); 2 Instituto de Ciencia y Tecnología de los Alimentos, Facultad de Ciencias Exactas, Físicas y Naturales, Universidad Nacional de Córdoba (ICTA-FCEFyN-UNC), Av. Vélez Sarsfield 1611, X5016GCA Córdoba, Argentina; 3 Chemical Engineering and Food Technology Department, Faculty of Science, Wine and Agrifood Research Institute (IVAGRO), University of Cadiz, 11519 Puerto Real, Spain; lourdes.casas@uca.es (L.C.-C.); casimiro.mantell@uca.es (C.M.-S.)

**Keywords:** olive leaves extract, polylactic acid, thermoplastic polyurethane, supercritical impregnation, antioxidant activity

## Abstract

The leaves of *Olea europaea* as agricultural waste represent a convenient source of antioxidants. In combination with supercritical CO_2_ (scCO_2_), assisted impregnation is an interesting strategy for the preparation of biomedical devices with specific bioactivity. For this purpose, 3D-printable filaments of thermoplastic polyurethane (TPU) and polylactic acid (PLA) were employed for the supercritical impregnation of ethanolic olive leaves extract (OLE) for biomedical application. The extraction of OLE was performed using pressurized liquids. The effect of pressure (100–400 bar), temperature (35–55 °C), and the polymer type on the OLE impregnation and the swelling degree were studied including a morphological analysis and the measurement of the final antioxidant activity. All the studied variables as well as their interactions showed significant effects on the OLE loading. Higher temperatures favored the OLE loading while the pressure presented opposite effects at values higher than 250 bar. Thus, the highest OLE loadings were achieved at 250 bar and 55 °C for both polymers. However, TPU showed c.a. 4 times higher OLE loading and antioxidant activity in comparison with PLA at the optimal conditions. To the best of our knowledge, this is the first report using TPU for the supercritical impregnation of a natural extract with bioactivity.

## 1. Introduction

Supercritical CO_2_ (scCO_2_) assisted impregnation has attracted growing attention in the last decade for the preparation of drug delivery systems and biomedical devices [1,2,3]. This promising technology offers several advantages over conventional methodologies mainly because the material is free of solvent at the end of the process, thus subsequent purification steps are not needed [1]. Moreover, it is a green technology that uses CO_2_ as solvent which is found abundantly in nature, and the operating conditions at which the supercritical state is obtained are easy to achieve (critical point at 31.1 °C and 73.8 bar) [4]. Supercritical solvents present intermediate properties between liquid and gaseous solvents, such as high densities as liquids and transport properties similar to gases. A remarkable feature of this technology is that these properties are tunable, allowing for the varying of the pressure and temperature [3].

Among the materials that have been used as platforms for impregnation, polymers are the preferred organic compounds, primarily due to their versatility [5,6,7]. They can be prepared by synthesis or extracted from natural resources, have good mechanical properties, can form solid or semisolid structures using the appropriate method, their chemical structure can be modified, the functional groups can interact with specific drugs, and some of them are biocompatible, which is essential for biomedical applications [8,9]. New forms of drug administration can be obtained using the 3D printing of biocompatible polymers to provide personalized medicine with therapeutic efficacy, safety, and precise control of drug dosing that can be adjusted to the patient’s compliance. This recent technology represents an innovative and auspicious strategy for biomaterials fabrication in the medical and pharmaceutical fields [10,11]. Among biocompatible polymers, polylactic acid (PLA) is one of the most employed for this purpose. PLA is a synthetic biopolymer belonging to the family of aliphatic polyesters that has been extensively used for the supercritical impregnation of bioactive compounds [12]. PLA foams have been employed for the supercritical impregnation of cinnamaldehyde [13], and thymol [14,15]; it was also employed for the loading of aspirin in film form [16] and as a particle it was used for nitrendipine [17], ketoprofen and carvone [18], diclofenac [19], dihydroquercetin and α-tocopheryl succinate [20].

The combination of the supercritical impregnation and the 3D printing technology could result in a promising strategy for the preparation of new delivery systems. However, considering the high temperatures employed in the printing process, it is essential that the bioactivity of the final polymer is not affected. Recently, 3D-printable filaments of PLA have been employed for the supercritical impregnation of bioactive compounds. Verano-Naranjo and coworkers evaluated the effect of pressure and temperature on the impregnation of ketoprofen into PLA filaments using 100–400 bar and 35–75 °C [21]. In a later work, they studied the mechanism of ketoprofen in in vitro release from the impregnated samples [22]. Rosales et al. studied the supercritical impregnation of PLA filaments with ethanolic mango leaves extract using different pressure, temperature, and extract concentration conditions [23]. Moreover, these authors evaluated the bioactivity of the polymer filament after the printing process, and the antioxidant activity was maintained to a great extent. These results encourage the research in this field, although a high antioxidant activity needs to be guaranteed before impregnation in order to minimize bioactivity whether for the change in the polymer structure after melting-printing or the slight loss of bioactivity. In this regard, this high bioactivity needs to be assessed through the study of the influence of the operational conditions on each raw material.

From a medical point of view, thermoplastic polyurethane (TPU) has been extensively used in biomedical devices (catheters, stents, orthopedics) [24,25,26,27] and has shown potential as a drug delivery system [9,24,28]. TPU is a copolymer composed of urethane moieties with alternating hard and soft blocks [25]. The hard segments impart mechanical strength and are formed by the isocyanate and the chain extenders, whereas the soft segments are composed of polyethers and provide flexibility [28,29]. The supercritical processing of TPU has mainly focused on the fabrication of foams using scCO_2_ as a blowing agent [30,31,32]. To the best of our knowledge, the supercritical impregnation of TPU 3D-printable filaments has not been reported, however, only a single report was found in the literature regarding the supercritical impregnation of TPU (in the film form) with model drugs. These authors impregnated TPU films with rhodamine B, fluorescein and 7-hydroxicoumarin at 150 bar and 40 °C for 4 h, reaching loadings of 1–4% with different drug release profiles [33].

Among the bioactive compounds, plant extracts and their main components have received much attention for many years. Many plant extracts have potential beneficial therapeutic effects which generally are a consequence of a synergistic activity among their bioactive components [34,35]. Currently, the request for the replacement of synthetic antioxidants by natural ones has been increasing. One of the richest sources of natural antioxidants is the olive tree (*Olea europaea*). The main constituents of the olive extract are polyphenols belonging to the families of secoroids like oleuropein, flavonols like luteolin-7-glucoside, and simple phenols represented by hydroxytyrosol [36]. The interest in olive oil production and consumption has expanded olive cultivation to regions and countries outside the Mediterranean Basin such as South America. In the southern hemisphere, the biggest olive cultivation area is located in Argentina [37]. Thus, the leaves of *Olea europaea* as by-products or agricultural waste represent a convenient source of antioxidants. The extraction of phytochemicals from plant materials can be performed using conventional methods (Soxhlet, maceration, reflux, etc.) or alternative methods like pressurized liquid extraction. This method is carried out at elevated temperature and pressure conditions and has the advantages of reducing the consumption of organic solvents, faster extraction times, higher extraction yields, and reproducibility [38,39]. The extraction of olive leaves extract (OLE) using pressurized liquid and the subsequent supercritical impregnation was previously performed into polyethylene terephthalate/polypropylene (PET/PP) films for active packaging in the food industry [36,40].

In the present work, the supercritical impregnation of OLE was performed into filaments of PLA and TPU for biomedical applications. The effect of different operating variables such as pressure, temperature, and the polymer type on the loading of OLE and the swelling degree were studied including the antioxidant activity of the impregnated filaments.

## 2. Materials and Methods

### 2.1. Materials

Pure transparent filaments of polylactic acid (PLA) and thermoplastic polyurethane (TPU) with 1.75 mm of nominal diameter were provided by Mundo Reader S.L. (Madrid, Spain) and GEEETECH (Shenzhen, China). Some thermal properties of the polymers provided by the manufacturer are included in Table 1. The *Olea europaea* leaves were provided by an olive oil producer (San José de Lora de Estepa Olivarera Sca Coop, Seville, Andalusia, Spain) and were dried at room temperature and stored in the dark prior to use.

Carbon dioxide (CO_2_, 99.99% purity) was purchased from Abelló Linde S.A. (Barcelona, Spain). The analytical grade solvents, ethanol, trichloromethane, dimethylsulfoxide, and the other reactants, i.e., sodium hydroxide, sodium chloride, potassium chloride, sodium phosphate monobasic, and dibasic, were obtained from Panreac AppliChem (Darmstadt, Germany).

The reactant 2,2-diphenyl-1-picrylhidrazil (DPPH) for the antioxidant assays was purchased from Sigma-Aldrich (Steinhelm, Germany).

### 2.2. Methods

#### 2.2.1. Enhanced Solvent Extraction of *Olea europaea* Leaves

The enhanced solvent extraction of *Olea europaea* leaves was performed following a previous procedure with slight modifications [36]. In brief, 195 g of dried and crushed leaves were weighed in a paper filter cartridge and introduced into a 1 L high-pressure vessel provided by Thar Technologies (Pittsburgh, PA, USA, SF500). The high-pressure system also includes temperature control, two high-pressure pumps (one for the supercritical CO_2_ and the other for the co-solvent), an automatic back-pressure regulator (BPR), and a cyclonic separator. The extractions were carried out in batch mode using a mixture of CO_2_ and ethanol (3% *v*/*v*) at 120 bar and 80 °C, for 3 h. The mixture was pumped at a flow rate of 10 g min^−1^ until the desired pressure was reached, and the depressurization was controlled at a flow rate of 2 bars^−1^. The final extract was stored at room temperature and protected from light.

The concentration of the *Olea europaea* leaves extract (OLE) was expressed as g·mL^−1^ and the extraction yield (%*Y*) was calculated according to Equation (1)

(1) $$\%Y=\frac{m_{d}}{m_{l}}\times 100$$

where *m_d_* is the mass of dry extract (g) and *m_l_* is the initial mass of leaves (g). The extraction yield was determined in triplicate.

#### 2.2.2. Supercritical Solvent Impregnation

**Figure 1:**
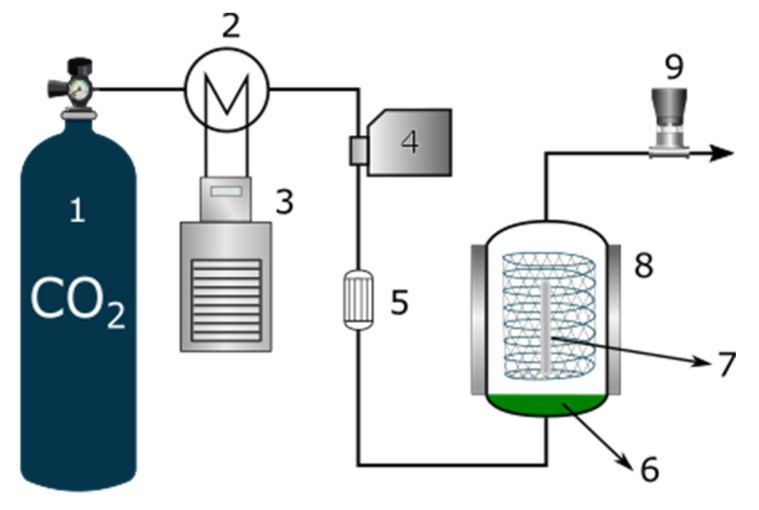
Schematic diagram of the supercritical solvent impregnation process. 1: CO_2_ cylinder; 2: condenser; 3: cold bath; 4: CO_2_ pump; 5: heat exchanger; 6: ethanolic OLE; 7: polymer filament; 8: high-pressure vessel; 9: automatic BPR.

The supercritical impregnation experiments were performed using high-pressure equipment provided by Thar Technologies (Pittsburgh, PA, USA, model SF100), and a schematic representation of it is depicted in Figure 1.

The experiments were carried out at different pressures (100, 250, and 400 bar), temperatures (35 and 55 °C), and with different polymers (PLA and TPU). The impregnation was conducted in batch mode using a 100 mL high-pressure vessel with a thermostatic jacket for controlling the temperature. 3 mL of ethanolic OLE (or 3 mL of pure ethanol for the swelling measurements) were introduced at the bottom of the vessel. A polymer filament of approx. 1.75 mm × 100 mm was vertically placed inside a metal cylinder covered with a metallic mesh and introduced into the vessel above the ethanolic OLE extract avoiding direct contact. Once the set temperature was reached inside the vessel, the CO_2_ was pumped at a flow rate of 10 g min^−1^ until the desired pressure was reached. The experiments were carried out for 1 h for OLE impregnation and 30 min for swelling tests. After that, the pressure was reduced at a rate of 2 bar·s^−1^. All impregnation runs were performed in duplicate.

#### 2.2.3. Swelling Degree

The swelling degree (%*S*) of the polymer filaments was determined after the treatment with pure scCO_2_, and the scCO_2_-ethanol mixture (3% *v*/*v*), and after the impregnation with ethanolic OLE. It was calculated according to Equation (2)

(2) $$\%S=\frac{v-v_{0}}{v_{0}}\times 100$$

where *v* and *v*_0_ represent the filament volume after and before the supercritical treatment. The filament volumes were calculated from the cylinder volume equation. The diameter and height of each filament were measured using a high-grade Vernier caliper. These determinations were performed in duplicate. A design of experiments was performed in order to determine the effect of some variables on the swelling of the polymer filaments (Section 2.2.5).

#### 2.2.4. OLE Loading (%OLE)

The loading of ethanolic OLE into the polymer filaments under supercritical conditions was determined with a spectrophotometric method using a UV-Vis spectrophotometer model Cary UV-Vis (Agilent Technologies, Santa Clara, CA, USA). For this purpose, 0.05 g of impregnated PLA or TPU were degraded employing 3 mL of trichloromethane and dimethyl sulfoxide, respectively. The absorbance of the resulting solutions was measured at 660 nm for both polymers, which is the wavelength corresponding to the OLE highest absorbance without the interference of any components produced by the degradation of the polymers. The amount of OLE impregnated into the polymers was calculated from the corresponding calibration curves (in the range of 0.4–4.0 mg mL^−1^) either in trichloromethane (y = 144.26x + 0.009, x = g mL^−1^, R^2^ = 0.9991) or dimethyl sulfoxide (y = 76.751x + 0.0159, x = g mL^−1^, R^2^ = 0.9945). The OLE loading was calculated according to Equation (3),

(3) $$\%OLE=\frac{m_{OLE}}{m_{POL}}\times 100$$

where *m_OLE_* is the amount of ethanolic OLE (g) impregnated into the polymers and *m_POL_* is the amount of polymer (g). The impregnation loadings were determined in duplicate.

#### 2.2.5. Design of Experiments

Two designs of experiments composed of three-factor and four-factor multilevel factorial design were performed in order to determine the best OLE impregnation conditions and the effect of some variables on the polymer swelling, respectively. For OLE loading, the studied process variables were the polymer, pressure, and temperature, whereas for the polymer swelling, the composition of the supercritical solvent was also included (Table 2).

The values of pressure and temperature were selected in order to cover a wide range of CO_2_ density (337.2–972.2 kg m^−3^). A total of 36 experiments were performed in duplicate, where 12 were performed to study the effect on the ethanolic OLE (%OLE) and 24 for the swelling of the polymer filaments (%S). The effect of each factor was statistically determined by the analysis of multifactorial variance (ANOVA) using the software Statgraphics© (StatPoint Technologies, Inc., Princeton, NJ, USA). Effects were considered significant for *p* < 0.05.

#### 2.2.6. Scanning Electron Microscopy

Non-treated (*in natura*), pressurized and impregnated filaments of PLA and TPU were observed under a Nova NanoSEM 450 microscope (FEI, Hillsboro, OR, USA) with the aim to visualize morphological changes and the feasibility of the OLE impregnation. The samples were prepared by cutting a small portion in the middle of the polymer filament using a cutting plier. The fragments were then coated with a thin layer of gold (10 nm) using a Cressington Sputter Coater model 208 HR from Cressington Scientific Instrument (Watford, UK) in order to improve their conductivity for better imaging.

#### 2.2.7. Antioxidant Activity

The antioxidant activity of the impregnated filaments was spectrophotometrically determined using the DPPH free radical scavenging assay, following a procedure reported in a previous contribution [23]. First, the antioxidant activity of the ethanolic OLE at different concentrations was determined. For this purpose, a 6 × 10^−5^ M DPPH ethanolic solution was prepared and stored in the dark prior to use. Then, 0.1 mL of OLE at different concentrations was mixed with 3.9 mL of DPPH ethanolic solution and left to react for 2 h protected from light. After that, the absorbance at 515 nm was measured using a UV-Vis spectrophotometer (Cary UV-Vis, Agilent Technologies, Santa Clara, CA, USA). The inhibition percentage (%*I*) was calculated according to Equation (4),

(4) $$\%I=\frac{A_{0}-A_{i}}{A_{0}}\times 100$$

where *A*_0_ is the absorbance at the initial point and *A_i_* is the absorbance after 2 h of reaction. A calibration curve was obtained from the plot %*I* vs. the initial concentration of OLE (in the range of 10–100 μg/mL) shown in Equation (5).

(5) $$y=-0.0145x^{2}+2.4501x+0.3236,\;R^{2}=0.997$$

The antioxidant activity of the impregnated filaments was determined as follows. A sample of impregnated filament (0.05 g) was submerged into 4 mL of 6 × 10^−5^ M DPPH ethanolic solution and the absorbance at 515 nm was measured after 2 h of reaction. The %*I* of the impregnated filaments was calculated using Equation (4), and Equation (5) was employed to determine the concentration of antioxidant components present in the impregnated filaments. All of these experiments were performed in duplicate.

## 3. Results

### 3.1. Polymer Swelling

**Figure 2:**
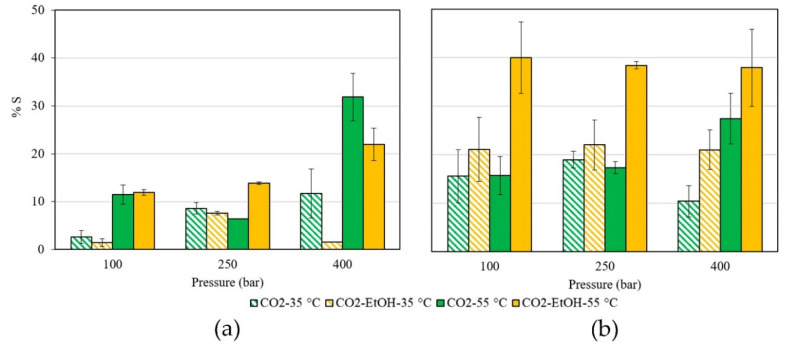
% Swelling of (**a**) PLA and (**b**) TPU filaments after supercritical treatment under 100, 250, and 400 bar using pure CO_2_ and a CO_2_:EtOH 3% *v*/*v* mixture as solvents, at 35 °C and 55 °C. The experiments were carried out for 30 min, in duplicate.

The permanent swelling of the studied polymers after the supercritical treatment was evidenced by changes in the filament volume. This phenomenon was evaluated using different pressures (100, 250, and 400 bar), temperatures (35 and 55 °C), and supercritical solvents (pure CO_2_ and a CO_2_:EtOH 3% *v*/*v* mixture). The results obtained for both polymers are shown in Figure 2.

Swelling values for PLA varied from 3 to 32% using pure scCO_2_ and 1–22% when the supercritical mixture CO_2_:ethanol was employed. These differences were not statistically significant (*p*-value 0.4466). The TPU swelling was higher than for PLA and, in contrast with PLA, it depended on the supercritical solvent. For TPU, the values ranged from 10–27% using pure scCO_2_, and they increased to 21–40% when ethanol was used as cosolvent. The ANOVA results evidenced that the type of polymer and the operation temperature, as well as the polymer-supercritical solvent interaction, showed significant effects on the polymer swelling (*p*-value < 0.05, Table S1, Supplementary Material).

**Figure 3:**
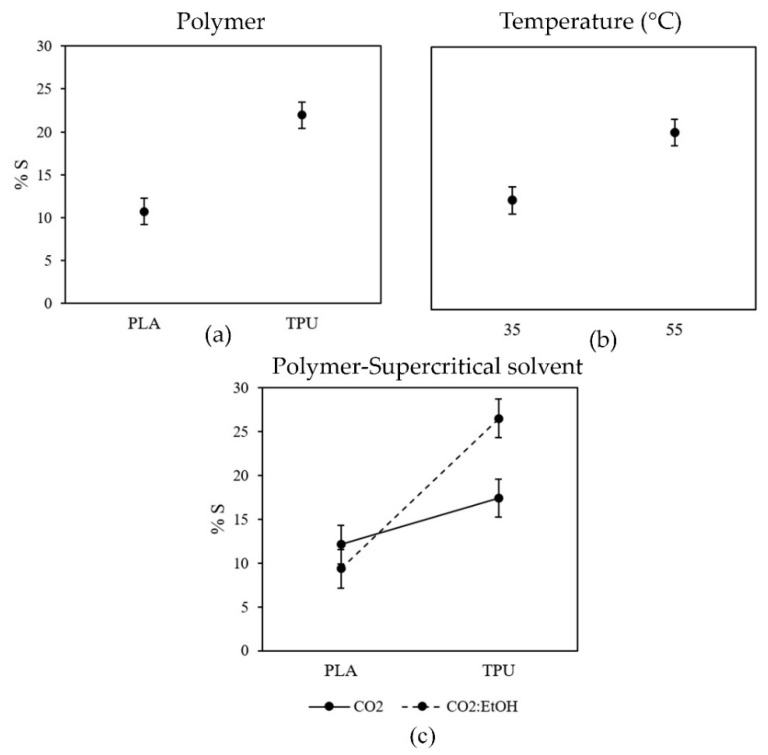
Plots of the multifactorial ANOVA analysis. Means plots for the statistically significant single factors: (**a**) polymer and (**b**) temperature, and interaction plots between two factors: (**c**) polymer-supercritical solvent, on the swelling of the polymer filaments (%S). All impregnation runs were performed for 30 min.

The effects of the studied variables on the polymer swelling are shown in Figure 3 (Data Table S2, Supplementary Material).

The single effects of the type of polymer and temperature were both positive, as can be seen in Figure 3a,b, respectively. Thus, TPU swelling was higher than for PLA and the results obtained at 55 °C were higher than at the lower temperature. However, the effect of the supercritical solvent was dependent on the type of polymer (Figure 3c, the non-parallel lines indicate an interaction between the factors). The addition of ethanol as cosolvent increased the swelling of TPU ca. 1.6 times (*p*-value 0.0302) although this effect was not significant for PLA (*p*-value 0.4466). The possible interactions between the supercritical solvent and the polymer employed are discussed in more detail in Section 4.1.

### 3.2. OLE Loading and Swelling Degree of the Impregnated Filaments

The extraction of *Olea europaea* leaves was performed using enhanced solvent extraction according to an earlier contribution, with slight modifications [36]. In the present work, the extraction yield was 13 ± 1% at 120 bar, 80 °C after 3 h with a final OLE concentration of 0.11 ± 0.01 g mL^−1^ of ethanolic solution.

**Figure 4:**
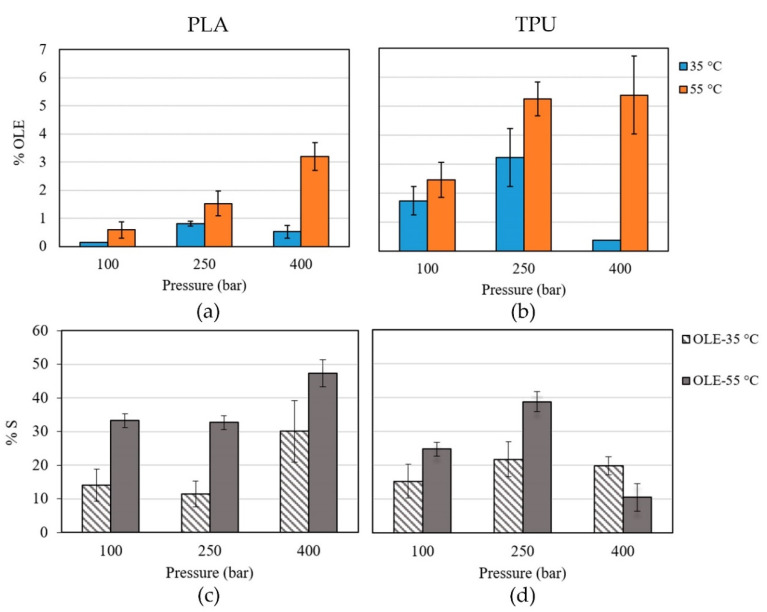
Ethanolic OLE loadings (%OLE) of (**a**) PLA and (**b**) TPU, and polymer swelling (%S) of (**c**) PLA and (**d**) TPU impregnated at 100, 250 and 400 bar, and 35–55 °C. All runs were performed in duplicate.

The OLE loading and swelling degree of polymer filaments were measured and the effect of process variables on these parameters was evaluated. The results obtained for OLE loading and polymer swelling are presented in Figure 4.

For PLA, the %OLE was in the range of 0.15–3.20% with a swelling degree of 11–47%, while for TPU these values were in the range of 0.39–5.39% and 11–39%, respectively.

All single factors (type of polymer, pressure, and temperature), and binary interactions presented significant effects on the %OLE (*p*-value < 0.05, Table S3, Supplementary Material). However, none of the studied operation parameters was significant on the swelling degree of the impregnated filaments (*p*-value > 0.05, Table S3, Supplementary Material). This finding may be related to the high experimental error involved in the swelling measurements.

**Figure 5:**
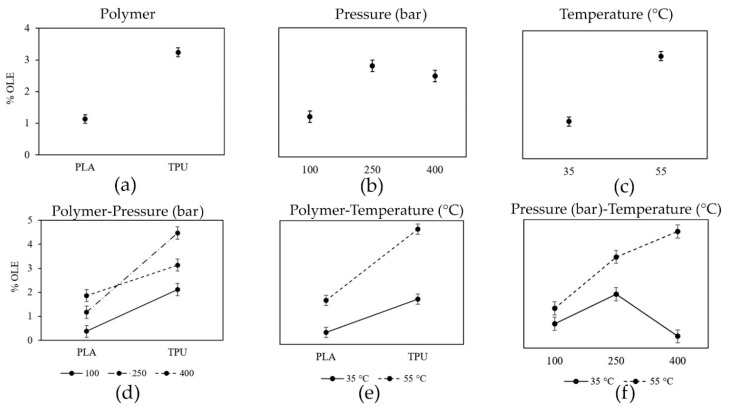
Plots of the multifactorial ANOVA analysis. Means plots for the statistically significant single factors: (**a**) polymer, (**b**) pressure and, (**c**) temperature; and interaction plots between two factors: (**d**) polymer-pressure, (**e**) polymer-temperature and (**f**) pressure-temperature, on the ethanolic OLE loading (%OLE). All impregnation runs were performed for 1 h. Non-parallel lines indicate an interaction between the factors.

Figure 5 shows the significant effect of the studied variables on the %OLE loading (Data Table S4, Supplementary Material).

Regarding the single factors, these effects were positive for the type of polymer and temperature, while for pressure the effect was positive from 100 to 250 bar and then remained constant. In this way, higher loadings were found using TPU regarding PLA (Figure 5a) and the ethanolic OLE loading increased with temperature (Figure 5c). However, the effect of pressure on OLE loading is more complex (Figure 5b). There is a positive effect from 100 to 250 bar but then the effect is invariable (from 250 to 400 bar, *p*-value 0.7757). Nevertheless, it depended on the type of polymer (Figure 5d). For PLA, an increase in the pressure also produced an increase in the OLE loading (*p*-value 0.0235), although, for TPU the OLE loading increased until the pressure reached 250 bar but after that pressure, the loading decreased (Figure 5d).

Figure 5e shows the effect of the interaction polymer-temperature. In general, the ethanolic OLE loading was higher at 55 °C than at 35 °C for both polymers, but, this effect is more pronounced for TPU than for PLA.

On the other hand, the particular effect of solvent density on the OLE loading can be analyzed in Figure 5f. Here, the OLE loading increased with the solvent density at 55 °C (337.2–906.85 kg m^−3^) but at 35 °C the OLE loading decreased when the pressure rose from 250 bar to 400 bar (i.e., from 901.05 to 972.2 kg m^−3^).

All the effects of the process variables (and their interactions) on the OLE loading are discussed in detail in Section 4.2.

The optimal conditions (among those studied in the present work) were 55 °C and 250 bar for both polymers. The samples impregnated at 55 °C were selected for the morphological characterization and the determination of the antioxidant activity due to the high OLE loadings.

### 3.3. Characterization of the Impregnated Polymer Filaments

#### 3.3.1. SEM Images

**Figure 6:**
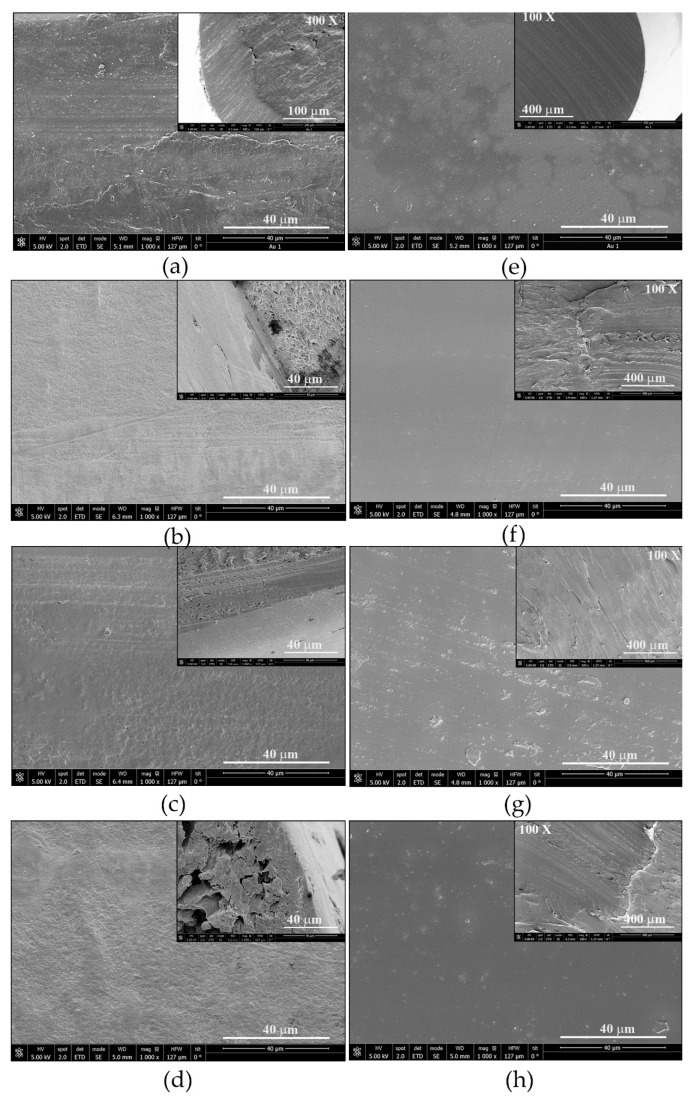
SEM images of longitudinal section of non-treated (*in natura*) filaments: (**a**) PLA and (**e**) TPU, and pressurized filaments of (**b**–**d**) PLA and (**f**–**h**) TPU after supercritical treatment with ethanol 3% *v*/*v* at 55 °C. (**b**,**f**) 100 bar; (**c**,**g**) 250 bar; (**d**,**h**) 400 bar. The upper right corner images are transversal sections of the polymer filaments. (Magnification ×1000 unless another value is indicated).

**Figure 7:**
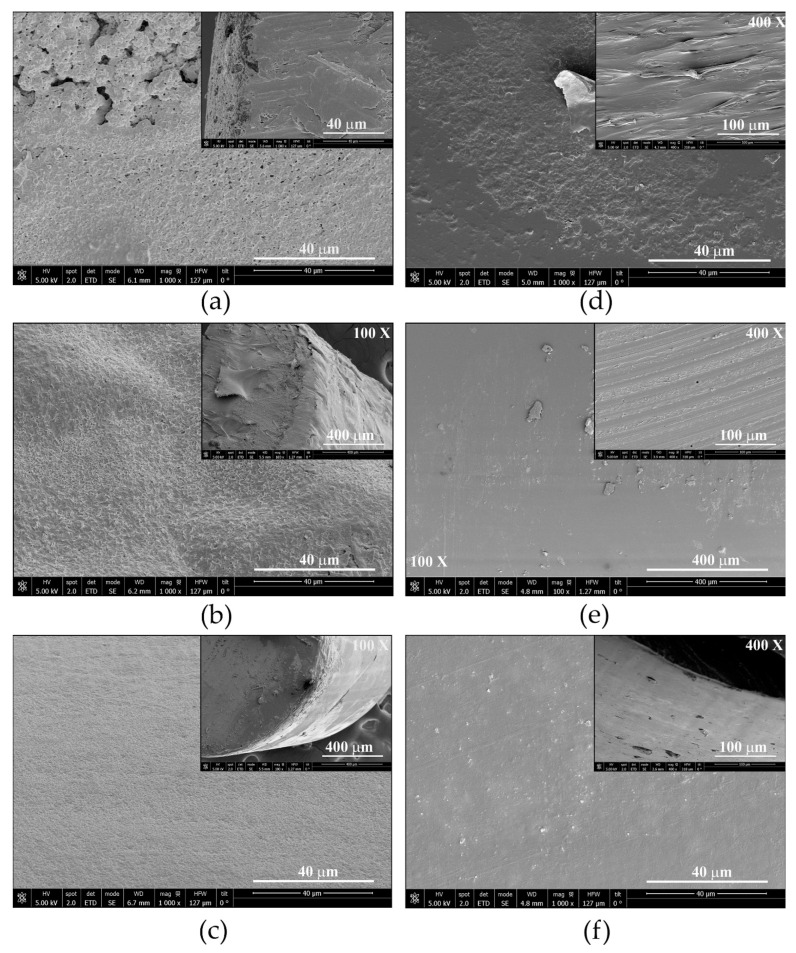
SEM images of longitudinal section of filaments impregnated with ethanolic OLE. (**a**–**c**) PLA and (**d**–**f**) TPU after supercritical treatment at 55 °C and (**a**,**d**) 100 bar; (**b**,**e**) 250 bar; (**c**,**f**) 400 bar. The upper right corner images are transversal sections of the polymer filaments. (Magnification ×1000 unless another value is indicated).

SEM images of the non-treated (*in natura*), pressurized and impregnated polymer filaments after the supercritical treatment are shown in Figure 6 (non-treated and non-impregnated) and Figure 7 (impregnated), respectively. These pictures include longitudinal and transversal (upper right corner images) section views of the polymer filaments. The morphological analysis is discussed further in Section 4.3.

#### 3.3.2. Antioxidant Activity

**Figure 8:**
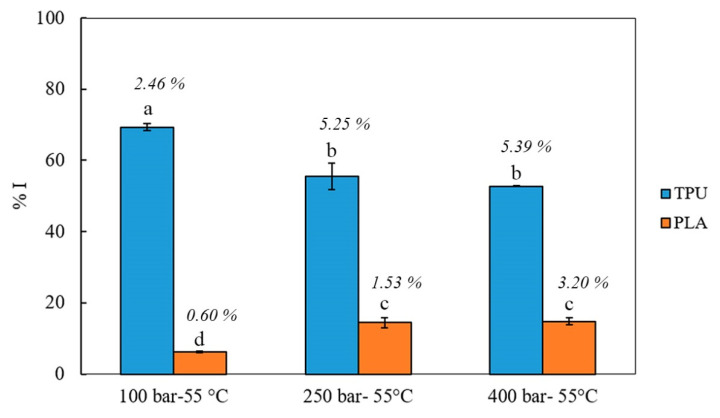
Percentages of oxidation inhibition (%I) for OLE-loaded polymer filaments impregnated under supercritical conditions (100, 250 and 400 bar and 55 °C, *n* = 2). Letters show statistical differences among samples (Tukey test, α = 0.05). Percentage values at the top of the columns indicate %OLE loading at each condition.

The antioxidant activity of the impregnated filaments obtained at 55 °C was determined following the DPPH assay. The results obtained are shown in Figure 8.

As can be seen, the antioxidant activity was considerably higher for TPU filaments than for PLA (*p*-value < 0.0001) reaching percentages of oxidation inhibition in the range of 52–69%, while for PLA these values were 6–15%. Moreover, the antioxidant loading for impregnated polymer filaments was calculated using Equation (5), and these values were 2–3 mg and 0.2–0.5 mg per g of polymer filament for TPU and PLA, respectively.

For TPU, the filaments impregnated at 100 bar reached the highest antioxidant capacity values (*p*-value 0.0363) although the higher OLE loadings were obtained at higher pressures (5.25 and 5.39% at 250 and 400 bar, respectively). For PLA, higher antioxidant capacities were obtained at higher pressures (*p*-value 0.016). On the other hand, there were not significant differences between 250 and 400 bar for both polymers (*p*-value > 0.05). These results suggest that the increase in solvent density at 55 °C did not necessarily produce higher antioxidant loading.

## 4. Discussion

The supercritical impregnation into polymeric materials involves three main steps. The first one is the dissolution of the solute in the supercritical solvent, the second one occurs when this solution diffuses inside the polymer matrix inducing the swelling of the polymer, and the last one results after the pressure drop that enables the recovery of the impregnated polymer free of solvent. For better understanding, the discussion of the results was divided into the following:

- polymer swelling (free of extract),
- ethanolic OLE loading into the polymer filaments and swelling after supercritical impregnation.

### 4.1. Polymer Swelling (Free of Extract)

**Figure 9:**
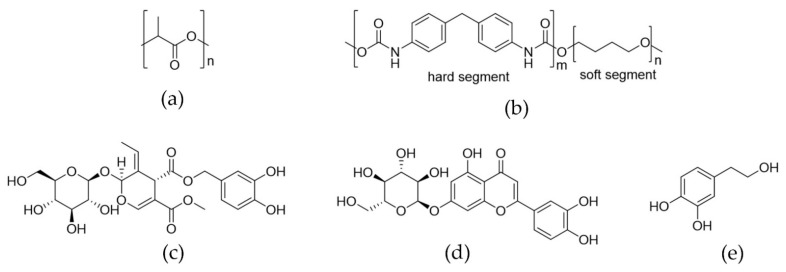
Chemical structure of the polymers (**a**) PLA and (**b**) TPU; and the main constituents of OLE extract (**c**) oleuropein, (**d**) luteolin-7-glucoside, and (**e**) hydroxytyrosol.

The sorption of the supercritical solvent into polymers usually results in their swelling induced by changes in the chain mobility and a consequent increase in the free volume. This phenomenon, known as plasticization, depends on the ability of the solvent molecules to interact with the functional groups of polymers. The chemical structures of the polymers employed are shown in Figure 9.

According to Section 3.1, TPU and PLA swelled after the supercritical treatment. These results indicate that both polymers can interact with the supercritical solvents. However, the swelling degree for TPU was higher than for PLA. The carbonyl groups present in the polyester units of PLA (Figure 9a) can interact with the electron-deficient carbon atom of CO_2_ via Lewis acid-base interaction [41,42]. However, stronger interactions may occur between TPU and CO_2_ via intermolecular hydrogen bonding between the N-H in the urethane groups of TPU (Figure 9b) and the oxygen in CO_2_ [41]. Moreover, the addition of ethanol as cosolvent increased the swelling of TPU, indicating that the presence of a polar cosolvent capable of forming hydrogen bonding improved the interaction between the fluid phase and the polymer urethane groups, inducing a higher swelling degree. This effect was not significant for PLA, possibly due to the lower affinity with polar solvents. Besides, the more highly ordered regions present in PLA in comparison with TPU may restrict the diffusion of the supercritical solvent inside this polymer and its swelling.

The temperature can also modify the swelling of the polymers altering the mobility of the polymer chains. Indeed, higher swelling degrees were found at higher temperatures (Figure 3b). The plasticization and consequent swelling of polymers induced by supercritical solvents can be a consequence of a glass transition temperature (Tg) reduction [43]. Above this temperature, the polymer changes from a rigid glassy material to soft rubbery material with enhanced molecular mobility [44]. TPU is a flexible polymer with a strong elastic behavior since it is typically above its Tg at room temperature [29]. In turn, PLA is a semicrystalline material with a Tg value higher than TPU (56–64 °C) (Table 1). The higher rigidity of PLA chains in comparison with TPU also contributes to limiting its swelling. Analyzing the effect of the temperature on the swelling degree of each polymer separately, the increase of temperature from 35 to 55 °C was significant for PLA (*p*-value 0.0145), in contrast with TPU (*p*-value 0.0936). The highest tested temperature is close to the PLA Tg, and considering that this temperature can decrease under supercritical conditions [45], it can be expected that the polymer may be fully plasticized at 55 °C, inducing a higher swelling than at 35 °C.

To the best of our knowledge, the scCO_2_-induced swelling behavior of TPU has not been studied, although some studies about the solubility and diffusivity of CO_2_ into this polymer have been previously performed [46]. Li et al. measured the foaming behavior of molten TPU at temperatures from 190–210 °C (Tm 170 °C) and pressures up to 193 bar and the results indicated a CO_2_ solubility in the range of 0.05–0.2 g g^−1^ with higher TPU foaming as the gas content increases [46]. Some reports of PLA swelling under similar conditions to those employed in this work have been found. Rosales et al. determined swelling values for PLA filaments ranging from 10 to 50% after 24 h of contact at 35–55 °C and 100–400 bar, varying the concentration of ethanol (1–3%). The highest swelling degree (50%) was found at 55 °C and 400 bar using 3% of ethanol as cosolvent. The authors suggested that two factors contributed to this swelling increase. On the one hand, the presence of ethanol may modify the internal structure of polymer chains favoring their mobility; and on the other hand, at higher pressure, the diffusion of CO_2_ inside the polymer could be facilitated [23]. In comparison with the present work, the higher PLA swelling observed by those authors may be due to the longer contact time employed (24 h vs. 30 min in the present work). Furthermore, the swelling of PLA in presence of pure scCO_2_ was also previously determined. Similar swelling values were reported by Verano-Naranjo et al. (4–25%) at 35–75 °C and 100–400 bar [21]. They observed an increase in the swelling degree at higher pressures and temperatures as a result of the higher scCO_2_ density and solvent diffusivity. Coutinho et al. measured a swelling degree of 14% for PLLA and 6% for LLDPE films using scCO_2_ at 80 °C and 300 bar after 3 h. The differences were attributed to the presence of ester groups in PLLA that can interact with CO_2_, increasing the chain mobility and swelling [16].

The polymer swelling under supercritical conditions could be favorable for the subsequent impregnation of a bioactive compound. Higher chain mobility and free volume improve the diffusion of the supercritical solvent-bioactive compound solution inside the polymer matrix increasing the impregnation loading. However, an uncontrolled swelling can lead to poor polymer mechanical properties. In this way, a balance between the loading and the swelling degree is necessary to obtain suitable materials for any proposed biomedical application.

### 4.2. OLE Loading and Swelling Degree of the Impregnated Filaments

The OLE extract employed in this work for the supercritical impregnation of TPU and PLA filaments was previously characterized [36]. Thus, the main constituents of this extract are polyphenols belonging to secoroids, flavonoids, and simple phenol families [36]. The loading values found in the present work are higher than those reported by other authors. Cejudo-Bastante et al. obtained OLE extract antioxidants loadings ranging from 0.05 to 0.25% into PET/PP films using supercritical impregnation under similar conditions [40]. Regarding the swelling degree, similar values were found by other authors in the supercritical impregnation of PLA filaments with mango leaves extract (10–40%). Although a statistical analysis was not performed, the authors observed higher swelling degrees at higher temperature and pressure [23]. The single report found for the supercritical impregnation of TPU films showed loading values similar to those obtained in the present work, from 1% for rhodamine B (hydrophobic model drug) to approximately 4% for 7-hydroxycoumarin (hydrophilic model drug) at 150 bar and 40 °C after 4 h [33]. Swelling measurements of TPU at the conditions employed in this work were not found in the literature.

The effect of the process variables on the swelling of the impregnated filaments was different from that observed for the polymer filaments free of extract. Despite the experimental error of these measurements, this result may also indicate that the addition of ethanolic OLE to the supercritical system changed the behavior of the supercritical solvent-polymer system. Thus, the polymer swelling depends not only on the type of polymer and its contact with scCO_2_, but also on the type of the bioactive compounds present in the impregnation process when it is a complex mixture such as natural extracts. In fact, these changes are evidenced by SEM, since TPU undergoes a more superficial impregnation than PLA, in which the higher penetration of the extract necessarily influences the final swelling. It is important to notice that the supercritical impregnation was performed at longer times in comparison with the swelling measurements (1 h vs. 30 min). In this sense, longer contact times and the presence of the ethanolic OLE governed the swelling of the polymers.

Regarding the supercritical loading of OLE, all single factors (type of polymer, pressure, and temperature) as well as their interactions, showed significant effects. Both polymers were loaded with OLE after the supercritical treatments, however, higher loadings were found for TPU than for PLA (Figure 5a). This phenomenon can be the result of two main effects, one of them is the high interaction between the bioactive compounds and the polymer matrix, and the other one is the ease of the supercritical solution to penetrate inside the polymer simultaneously with polymer swelling. The analysis of the possible interactions between the extract components and the polymer is quite difficult considering the complexity of the extract composition. Nevertheless, the functionalities of the main constituents will be considered for the analysis. According to a previous report, the 90% of the total olive extract polyphenols (obtained using the same procedure) are the hydrophilic species oleuropein, luteolin-7-glucoside and hydroxytyrosol (Figure 9c–e, respectively) [36]. All of these compounds have in common the presence of many hydroxyl groups able to interact via H-bonding with the carbonyl groups of the urethane and ester groups of TPU and PLA, respectively, promoting the loading. However, the presence of -NH groups in TPU (which are absent in PLA) favors the formation of H-bond interactions with the carbonyl groups of oleuropein and luteolin-7-glucoside, inducing higher loadings values in this polymer. Interactions like π-stacking can also occur between the aromatic rings of the main OLE components and the hard segments of TPU. Similar suggestions were proposed by Zhang and coworkers between the aromatic structure of rhodamine B and fluorescein and the hard blocks of TPU [33]. Interactions through carbonyl moieties in OLE compounds were evidenced by Cejudo-Bastante and coworkers on the surface of PET layers [40]. The occurrence of interactions between the hydroxyl group of thymol and the carbonyl groups of polyesters were observed in PLA and PLGA foams [14], and in surgical sutures made of caprolactone and glycolide copolymer [47]. Mosquera et al. attributed higher eugenol loadings into polyamide 6 to the interaction via hydrogen-bonding with the OH and ether groups of eugenol [48]. Furthermore, not only H-bond interactions are significant for the polymer affinity but also the high hydrophilic character of the polyphenols that may displace the partition toward the TPU (due to its polarity) instead of the supercritical fluid. Previously, higher loadings were found in TPU films at 150 bar and 40 °C for the hydrophilic model drug than for the hydrophobic one after the supercritical impregnation [33]. Additionally, the presence of ethanol as cosolvent could improve the affinity of polyphenols not only with the polymer (i.e., positive effect on the OLE loading) but also with the fluid phase (negative effect on the OLE loading). However, the positive effect could prevail since the presence of ethanol improves the affinity between the fluid phase and the polymer facilitating the diffusion inside the polymer and thus improving the loading of OLE. The possible interactions between TPU and the cosolvent were discussed in Section 4.1.

The temperature was another single factor that showed a positive effect on the ethanolic OLE loading which increased with it (Figure 5c). Higher temperatures can decrease the density of scCO_2_ and alter the structure of the polymers. The reduction of scCO_2_ density favors the partition of OLE compounds through the polymer matrix by decreasing their solubility in the fluid phase. Besides, the mobility of the polymer chains at 55 °C is higher than at 35 °C specifically for thermoplastic polymers such as TPU and for polymers with a glass transition temperature near to the operating temperature, such as PLA. Similar effects were found by Rosales et al. in the supercritical impregnation of mango leaf extract into PLA filaments, where higher loadings were obtained at 55 °C than at 35 °C [23]. Also, Verano-Naranjo and coworkers observed higher loadings of ketoprofen inside PLA at 75 °C than at 35 °C as result of the combination between the higher swelling of the polymer at that temperature and the decrease in the scCO_2_ density [21]. On the contrary, the temperature (35 and 55 °C) did not affect the supercritical loading of OLE into PET/PP films after 1 h at 100 and 400 bar [40], confirming the hypothesis that the effect of temperature on the loading of OLE is mainly governed by the type of polymer.

According to Figure 5e, the effect of the temperature on the OLE loading was more pronounced for TPU than for PLA. It is possible that the highly ordered regions of PLA in comparison with TPU limits the diffusion of the supercritical solution even at a higher temperature, restricting its loading capacity.

The general effect of pressure on the OLE loading was variable. The %OLE increased when the pressure rise from 100 to 250 bar, but after that pressure, it remained constant (Figure 5b). However, this effect depended on the type of polymer (Figure 5d), as mentioned before. A significant increase in the OLE loading was produced with the pressure when PLA filaments were employed. The scCO_2_ density increases at higher pressures and isothermal conditions, improving the solvent power. Thus, an increment in the pressure may promote the solubility of the polyphenols in the fluid phase favoring the mass transfer [36,40].

The behavior observed for TPU, as pressure increases, was different. For this polymer, the OLE loading increased until the pressure reached 250 bar (Figure 5d) but after that, the loading decreased. At higher pressure values, the negative effect on the OLE loading could prevail, since the partitioning becomes more favorable for the supercritical solvent than for the polymer decreasing the loading.

The particular effect of solvent density (i.e., the interaction pressure-temperature) on the OLE loading can be deducted from Figure 5f. Here, the OLE loading increased when the solvent density rose from 337.2 to 906.85 kg m^−3^ (corresponding to 100 and 400 bar at 55 °C, respectively), but higher solvent densities like those achieved at 35 °C decreased the OLE loading (901.05–972.2 kg m^−3^ corresponding to 250 and 400 bar, respectively). This phenomenon may indicate that higher densities favor the partition to the fluid phase and compromise the loading of OLE extract inside the polymers, as previously mentioned. A negative effect of CO_2_ density on the loading of mango leaf extract into PLA filaments was also previously observed [23]. Besides, Milovanovic et al. reported that an increase in the solvent density (273–630 kg m^−3^) did not necessarily increase the thymol loadings in PLA foams [14].

In this way, the optimal conditions (55 °C and 250 bar for both polymers) were selected according to the highest OLE loading at the lowest energy cost and CO_2_ consumption, as well as the minimal effect on the polymer integrity under the supercritical conditions.

### 4.3. Characterization of the Impregnated Polymer Filaments

#### 4.3.1. Morphological Analysis

In general, some of the characteristic imperfections of the materials as well as those caused by the cutting of the sample can be observed in Figure 6 and Figure 7, especially for the images of the transversal sections (upper right corner).

Although the statistical analysis did not show a significant effect of the pressure on the swelling of the polymers, a general effect of this variable could be observed from the SEM images of the non-impregnated filaments (Figure 6). Here, rough surfaces are observed, and this effect was more remarkable in PLA than in TPU filaments (Figure 6). The roughness observed in the longitudinal section of PLA increases with the pressure (Figure 6b–d). Furthermore, an increase in the formation of holes or channels in this polymer is observed from the transversal section (upper right corner images) when pressure increases, which are absent in the non-treated PLA filament (Figure 6a). These pictures demonstrate that PLA is affected by the supercritical treatment and suggest that not only the surface has been altered but also that its internal arrangement has changed. Such structural changes were not clearly observed in the non-impregnated TPU filaments (Figure 6f–h). Verano-Naranjo and co-workers observed similar structural changes in PLA filaments as those observed in the present work after the supercritical treatment using 75 °C and 250 bar [21].

The OLE impregnated PLA filaments shows also show rough surfaces with the deposition of a dense layer on their surfaces, which is principally visible in the transversal sections (Figure 7a–c). On the other hand, the longitudinal sections of TPU filaments (Figure 7d–f) show more irregular surfaces in comparison with non-impregnated TPU filaments (Figure 6f–h). The surface of the TPU filaments was more regular than for PLA despite the higher loadings and swelling degree. These results may suggest the higher physical stability of TPU after the supercritical treatment in comparison with PLA. Rosales et al. observed irregular surfaces with bulges and pores in PLA filaments after the impregnation of mango leaves extract at 100 bar and 35 °C, resulting in the swelling of the polymer in the presence of the extract [23].

#### 4.3.2. Antioxidant Activity

Free radicals at pathologic levels induce oxidative damage in biomolecules (e.g., lipids, proteins, amino acids, and DNA), and this damage could cause cell injury and the subsequent development of different types of pathologies such as cardiovascular, neurodegenerative and autoimmune diseases [49,50]. The DPPH assay is a practical and useful method to measure the in vitro antioxidant activity of pure compounds and plant extracts. In this reaction, the odd electron of the nitrogen atom of the DPPH radical is reduced by receiving a hydrogen atom from antioxidant compounds resulting in the solution color decrease [51].

The antioxidant activity of the impregnated filaments was considerably higher for TPU filaments than for PLA (Figure 8). These results indicate that the amount of antioxidant components present in the impregnated TPU filaments are highly associated with the higher OLE loadings reached using this polymer. In fact, the antioxidant loading for impregnated TPU filaments was ca. 4–10 times higher than for PLA. Under similar conditions, Cejudo-Bastante et al. obtained OLE antioxidant loadings of approximately 1 mg and 2 mg per g of PET/PP films at 100 bar and 400 bar, respectively, at the same temperature [40]. When PLA filaments were impregnated with 3% of ethanolic mango leaves extract, the percentage of oxidation inhibition was almost 20% at 100 bar and 45% at 400 bar and 55 °C for both [23]. The different values of antioxidant activity observed for PLA may be related to the higher affinity with mango polyphenols regarding those present in the olive leaves extract.

The differences observed between the higher antioxidant loading and percentage of OLE loading in both polymers confirm the complexity of the equilibrium reached under supercritical conditions between the extract components, the polymer matrices, and the supercritical solvent. The mixture of polyphenols in the extract that have antioxidant activity may present different selectivity under the conditions employed in this work. In this way, the effect of process variables on the antioxidant loading is different from the effect on the OLE loading.

## 5. Conclusions

The supercritical impregnation of ethanolic *Olea europaea* extract was successfully performed in thermoplastic polyurethane and polylactic acid filaments. All the studied process variables (temperature, pressure and type of polymer) showed significant effects on the OLE loading. This result suggests the high complexity of the supercritical impregnation of natural extracts into biocompatible polymers. Higher temperatures favored the OLE loading by improving the diffusion of the fluid phase inside the polymers, while the pressure presented opposite effects at values higher than 250 bar. Thus, the highest OLE loadings were achieved at 250 bar and 55 °C for both polymers.

Considering the type of polymer, thermoplastic polyurethane showed a higher swelling degree (without visible physical damage), a *c.a.* four times higher OLE loading and antioxidant activity in comparison with polylactic acid at the optimal conditions (55 °C and 250 bar). To the best of our knowledge, this is the first report using thermoplastic polyurethane for the supercritical impregnation of a natural extract with bioactivity.

However, more studies are needed to evaluate the release mechanism of the antioxidant compounds into simulated physiological medium and of the mechanical properties of the impregnated filaments after the supercritical treatment for its application in the biomaterials fabrication for the medical and pharmaceutical fields. In addition, in vitro and in vivo assays are also required to ensure the cellular viability and the in vivo performance of the impregnated filaments.

## Figures and Tables

**Table 1:** Thermal properties of the polymers.

Thermal Property	PLA	TPU	Standard Test
Melting temperature (°C)	145–160	165	ASTM D3418
Glass transition temperature (°C)	56–64	−25	ASTM D3418

**Table 2:** Design of experiments for the polymer swelling degree (%S) and ethanolic OLE loading (%OLE).

Experiment	Fixed Parameters		Studied Variables		Response Variables
Swelling	Pressurization rate	10 g min^−1^	Polymer	PLA, TPU	%S
	Contact time	30 min	Pressure (bar)	100, 250, 400	%S
	Polymer filament dimensions	1.75 mm × 100 mm	Temperature (°C)	35, 55	%S
	Depressurization rate	2 bar s^−1^	Supercritical solvent	Pure CO_2_, CO_2_:EtOH 3% *v*/*v*	%S
Supercritical impregnation	Pressurization rate	10 g min^−1^	Polymer	PLA, TPU	%OLE, %S
	Contact time	1 h	Pressure (bar)	100, 250, 400	%OLE, %S
	Polymer filament dimensions	1.75 mm × 100 mm	Temperature (°C)	35, 55	%OLE, %S
	Depressurization rate	2 bar s^−1^	-	-	-
	OLE concentration (ethanolic solution)	0.11 ± 0.01 g mL^−1^	-	-	-

## Data Availability

Data is contained within the article and Supplementary Material.

## Supplementary Materials

The following supporting information can be downloaded at: https://www.mdpi.com/article/10.3390/antiox11061170/s1, Table S1: ANOVA testing the effects of process variables and their interactions on the polymer swelling (%S) for the fractional design model. Effects were considered significant for *p* < 0.05; Table S2: The least-square mean values for % Swelling with a 95.0% confidence interval; Table S3: ANOVA testing the effects of process variables and their interactions on the ethanolic OLE loading (%OLE) and swelling degree of the impregnated samples (%S) for the fractional design model. Effects were considered significant for *p* < 0.05; Table S4. The least-square mean values for % OLE with a 95.0% confidence interval.
