# Supplementary material for: Screening of the Supercritical Impregnation of Olea europaea Leaves Extract into Filaments of Thermoplastic Polyurethane (TPU) and Polylactic Acid (PLA) Intended for Biomedical Applications
Source: Antioxidants (Basel). 2022 Jun 14;11(6):1170. doi: 10.3390/antiox11061170 (PMC9219857; doi:10.3390/antiox11061170)
Supplement: Supplementary file 1 [file antioxidants-11-01170-s001.zip › antioxidants-1742977-supplementary.pdf]

Table S1. ANOVA testing the effects of process variables and their interactions on the polymer swelling (% S) for the fractional design model. Effects were considered significant for $p < 0.05$.

Factor	DF	SS	MS	F	p-value
A: Polymer	1	1510.12	1510.12	26.56	**<0.0001**
B: Pressure (bar)	2	96.38	48.19	0.85	0.4376
C: Temperature (°C)	1	721.18	721.18	12.68	**0.0011**
D: Supercritical solvent	1	121.09	121.09	2.13	0.1539
AB	2	326.51	163.26	2.87	0.0709
AC	1	7.24	7.24	0.13	0.7234
AD	1	422.17	422.17	7.43	**0.0102**
BC	2	152.49	76.25	1.36	0.2755
BD	2	185.56	92.78	1.63	0.2110
CD	1	116.61	116.61	2.05	0.1615

DF: degrees of freedom. SS: sum of squares. MS: mean square.

Table S2. The least-square mean values for % Swelling with a 95.0% confidence interval

Level	Count	Mean	Stnd. Error	Lower Limit	Upper Limit
GRAND MEAN	48	16.3307			
Polymer					
-1	24	10.7217	1.53917	7.59023	13.8532
1	24	21.9397	1.53917	18.8082	25.0712
Pressure					
100	16	14.5902	1.88509	10.755	18.4255
250	16	16.3408	1.88509	12.5055	20.176
400	16	18.0611	1.88509	14.2258	21.8963
Temperature					
35	24	12.4545	1.53917	9.32308	15.586
55	24	20.2068	1.53917	17.0754	23.3383
Solvent					
-1	24	14.7424	1.53917	11.6109	17.8739
1	24	17.919	1.53917	14.7875	21.0504
Polymer by Pressure					
-1;100	8	6.93868	2.66591	1.51482	12.3625
-1;250	8	9.09326	2.66591	3.66941	14.5171
-1;400	8	16.1331	2.66591	10.7093	21.557
1;100	8	22.2418	2.66591	16.818	27.6657
1;250	8	23.5883	2.66591	18.1644	29.0121
1;400	8	19.989	2.66591	14.5652	25.4129
Polymer by Temperature					
-1;35	12	6.45702	2.17671	2.02846	10.8856
-1;55	12	14.9864	2.17671	10.5578	19.4149
1;35	12	18.4521	2.17671	14.0235	22.8806
1;55	12	25.4273	2.17671	20.9988	29.8559
Polymer by solvent					
-1;-1	12	12.0991	2.17671	7.67051	16.5276
-1;1	12	9.34431	2.17671	4.91575	13.7729
1;-1	12	17.3858	2.17671	12.9572	21.8143
1;1	12	26.4936	2.17671	22.0651	30.9222
Pressure by Temperature					
100;35	8	11.1942	2.66591	5.77039	16.6181

100;55	8	17.9863	2.66591	12.5624	23.4101
250;35	8	14.3675	2.66591	8.94369	19.7914
250;55	8	18.314	2.66591	12.8901	23.7378
400;35	8	11.8018	2.66591	6.37799	17.2257
400;55	8	24.3203	2.66591	18.8964	29.7441
Pressure by solvent					
100;-1	8	12.9918	2.66591	7.56792	18.4156
100;1	8	16.1887	2.66591	10.7649	21.6126
250;-1	8	12.3495	2.66591	6.92569	17.7734
250;1	8	20.332	2.66591	14.9081	25.7558
400;-1	8	18.8859	2.66591	13.4621	24.3098
400;1	8	17.2362	2.66591	11.8124	22.6601
Temperature by solvent					
35;-1	12	12.4249	2.17671	7.99635	16.8535
35;1	12	12.4842	2.17671	8.05562	16.9127
55;-1	12	17.0599	2.17671	12.6313	21.4885
55;1	12	23.3538	2.17671	18.9252	27.7823

Table S3. ANOVA testing the effects of process variables and their interactions on the ethanolic OLE loading (% OLE) and swelling degree of the impregnated samples (% S) for the fractional design model. Effects were considered significant for $p < 0.05$.

Experiment	Factor	DF	SS	MS	F	*p*-value
% OLE	A: Polymer	1	26.67	26.67	105.03	**<0.0001**
	B: Pressure (bar)	2	11.09	5.55	21.85	**<0.0001**
	C: Temperature (°C)	1	25.12	25.12	98.94	**<0.0001**
	AB	2	4.48	2.24	8.83	**0.0033**
	AC	1	3.52	3.52	13.85	**0.0023**
	BC	2	13.21	6.61	26.02	**<0.0001**
% S	A: Polymer	1	38.70	39.70	0.36	0.5585
	B: Pressure (bar)	2	85.50	42.75	0.40	0.6798
	C: Temperature (°C)	1	466.38	466.38	4.33	0.0563
	AB	2	425.52	212.76	1.97	0.1756
	AC	1	92.20	92.20	0.86	0.3706
	BC	2	627.31	313.67	2.91	0.0877

DF: degrees of freedom. SS: sum of squares. MS: mean square.

Table S4. The least-square mean values for % OLE with a 95.0% confidence interval

Level	Count	Mean	Stnd. Error	Lower Limit	Upper Limit
GRAND MEAN	24	2.18805			
Polymer					
-1	12	1.1339	0.145466	0.821906	1.44589
1	12	3.2422	0.145466	2.93021	3.5542
Pressure					
100	8	1.24468	0.178158	0.862562	1.62679
250	8	2.82088	0.178158	2.43876	3.20299
400	8	2.49861	0.178158	2.11649	2.88072
Temperature					

35	12	1.16492	0.145466	0.852923	1.47691
55	12	3.21119	0.145466	2.89919	3.52318
Polymer by Pressure					
-1;100	4	0.3718	0.251954	-0.168589	0.912189
-1;250	4	1.17058	0.251954	0.630186	1.71096
-1;400	4	1.85933	0.251954	1.31894	2.39971
1;100	4	2.11755	0.251954	1.57716	2.65794
1;250	4	4.47117	0.251954	3.93079	5.01156
1;400	4	3.13789	0.251954	2.5975	3.67828
Polymer by Temperature					
-1;35	6	0.4935	0.20572	0.0522744	0.934726
-1;55	6	1.7743	0.20572	1.33307	2.21553
1;35	6	1.83633	0.20572	1.39511	2.27756
1;55	6	4.64808	0.20572	4.20685	5.0893
Pressure by Temperature					
100;35	4	0.944475	0.251954	0.404086	1.48486
100;55	4	1.54487	0.251954	1.00449	2.08526
250;35	4	2.09503	0.251954	1.55464	2.63541
250;55	4	3.54673	0.251954	3.00634	4.08711
400;35	4	0.45525	0.251954	-0.0851388	0.995639
400;55	4	4.54196	0.251954	4.00158	5.08235
